# Pathogenicity Classification of *TARDBP* Variants of Uncertain Significance: An Integrative Clinical Characterization and Functional Validation

**DOI:** 10.3390/cells15141232

**Published:** 2026-07-08

**Authors:** Chao-Sen Yang, Yuan Ma, Jia-Li Xie, Xin-Yan Lou, Yong-Ting Lv, Tan-Xia Wu, Hai-Feng Xu, Sheng-Mei Zou, Zhi-Ying Wu, Hong-Fu Li

**Affiliations:** 1 Department of Medical Genetics and Center for Rare Diseases, Second Affiliated Hospital, Zhejiang University School of Medicine, and Zhejiang Key Laboratory of Rare Diseases for Precision Medicine and Clinical Translation, Hangzhou 310002, China; yangchaosen@zju.edu.cn (C.-S.Y.); yuanma2024@zju.edu.cn (Y.M.); xjiali1223@zju.edu.cn (J.-L.X.); 22318456@zju.edu.cn (Y.-T.L.); 12518443@zju.edu.cn (T.-X.W.); haifengxu@zju.edu.cn (H.-F.X.); shengmeizou@zju.edu.cn (S.-M.Z.); 2 Nanhu Brain-Computer Interface Institute, Hangzhou 310002, China; 3 Department of Neurology, Second Affiliated Hospital, Zhejiang University School of Medicine, Hangzhou 310002, China; 4 College of Laboratory Medicine (College of Life Sciences), Wenzhou Medical University, Wenzhou 325035, China; louxinyan216@gmail.com

**Keywords:** *TARDBP*, pathogenicity, amyotrophic lateral sclerosis, clinical characterization, functional validation

## Abstract

**Highlights:**

**What are the main findings?**

- 28 TAR DNA binding protein (*TARDBP*) variants of uncertain significance (VUS) were functionally evaluated; 22 exhibited functional defects, and 12 were reclassified as likely pathogenic per ACMG/ClinGen guidelines.
- Pathogenic *TARDBP* missense variants cluster in the TDP-43 C-terminal domain; exon 6 variants are associated with earlier disease onset, accompanied by marked phenotypic heterogeneity and incomplete penetrance.

**What is the implication of the main finding?**

- This study expands the pathogenic *TARDBP* mutation spectrum and delineates the natural history of affected patients, providing support for clinical genetic counseling and ALS risk assessment.
- It provides preliminary evidence validating patient-derived fibroblasts as a viable cellular model for functional assays, laying a cellular foundation for future functional investigations of *TARDBP*-related ALS.

**Abstract:**

TAR DNA binding protein (*TARDBP*) is one of the major causative genes of amyotrophic lateral sclerosis (ALS), which drives disease progression through both gain-of-toxicity (GOT) and loss-of-function (LOF) mechanisms. The mutant TDP-43 exhibits aberrant nucleocytoplasmic distribution and forms cytotoxic hyperphosphorylated aggregates, a process that can be robustly recapitulated in vitro. Thus, functional assays in cell lines serve as a reliable metric for the pathogenicity classification of *TARDBP* variants. In this study, we performed in vitro experiments to classify the pathogenicity of 28 *TARDBP* variants of uncertain significance (VUS) among the 172 previously reported *TARDBP* variants. 22 of these VUS were determined to be functionally abnormal, of which 12 could be further classified as likely pathogenic (LP) variants according to American College of Medical Genetics (ACMG) and the ClinGen Sequence Variant Interpretation (SVI) Working Group guidelines. We also summarized the clinical characteristics of 35 ALS patients carrying 12 variants in the *TARDBP* gene. Pathogenic missense variants were predominantly clustered in the C-terminal domain (CTD) of *TARDBP*. Variants in *TARDBP* exon 6 may lead to an earlier age at onset. ALS caused by *TARDBP* mutations exhibits marked phenotypic heterogeneity, along with incomplete penetrance in carriers. Patient-derived primary skin fibroblasts serve as a feasible cellular model for the functional assessment of variant pathogenicity. Our findings expand the *TARDBP* mutation spectrum, and provides a preliminary basis for preclinical research on *TARDBP*-targeted therapies for ALS.

## 1. Introduction

Amyotrophic lateral sclerosis (ALS) is a fatal neurodegenerative disorder characterized by the progressive death of motor neurons, with typical clinical manifestations of limb weakness and generalized muscle atrophy [1,2]. Most ALS cases are sporadic, while approximately 5–10% are familial forms with a clear family history and a higher yield of identifiable pathogenic mutations [3]. Among the over 40 characterized causative genes for ALS [4], the most common genetic causes include *C9orf72*, *SOD1*, *FUS,* and *TARDBP* [5,6,7,8]. Pathogenic variants in these genes can also be detected in a subset of apparently sporadic cases. The TDP-43 protein, which is encoded by the *TARDBP* gene, is widely recognized as a key player in the pathogenic mechanisms of ALS.

**Figure 1:**
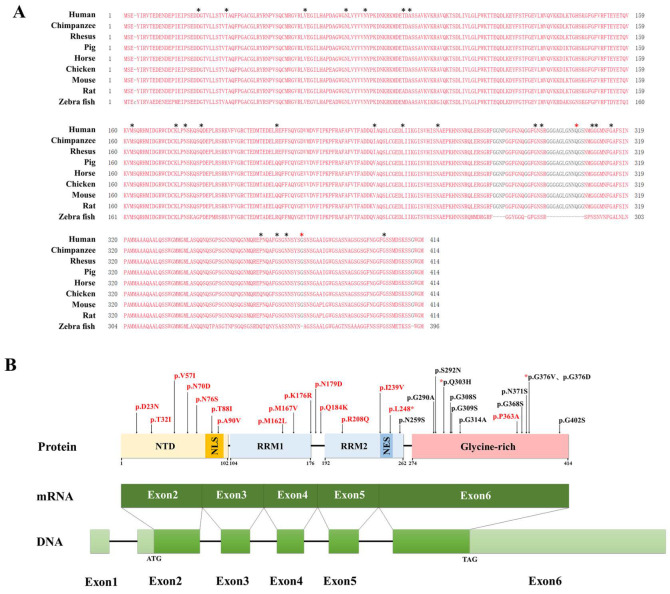
Schematic diagram of the *TARDBP* gene structure and its 28 variants of uncertain significance. (**A**) Amino acid sequence alignment of *TARDBP* among nine different vertebrate species. The 28 *TARDBP* variant loci included in this study are marked with asterisks: red asterisks indicate loci with low conservation, and black asterisks indicate loci with high conservation. (**B**) The *TARDBP* gene (NM_007375.4) consists of six exons with a full length of 1245 bp, and no distinct mutation hotspots were identified. The locations of all variant sites on the TDP-43 protein are annotated in the diagram. Variants annotated as likely pathogenic are labeled in black, while variants of uncertain significance are labeled in red. Red asterisks before variant loci represent low cross-species conservation, and variants without asterisks indicate high conservation. NTD, N-terminal domain; NLS, nuclear localization signal; RRM1, RNA recognition motif 1; NES, nuclear export signal.

TDP-43 is a nuclear RNA/DNA-binding protein composed of 414 amino acids [9]. TDP-43 consists of an N-terminal domain (NTD), two DNA/RNA-binding domains (RRM1 and RRM2), and a C-terminal domain (CTD) [10,11] (Figure 1B). TDP-43 is predominantly localized in the nucleus, where its nuclear localization signal (NLS) and nuclear export signal (NES) mediate its shuttling between the nucleus and cytoplasm via nucleocytoplasmic transport [12], thereby participating in the regulation of multiple aspects of RNA processing. The NTD and RRM1/RRM2 regions are highly conserved across different species (Figure 1A). The CTD is also referred to as a prion-like domain (PLD) [13]. The CTD exhibits low sequence complexity and intrinsic structural disorder, resulting in considerable divergence among species (Figure 1A). Pathogenic mutations in *TARDBP* are mainly located in the CTD [14,15].

TDP-43 is predominantly nuclear-localized, and regulates RNA splicing, transport and stability to maintain normal cellular function. [10]. Mutant TDP-43 protein encoded by pathogenic *TARDBP* gene variants forms toxic and insoluble aggregates in the cytoplasm, leading to motor neuron toxicity [14,16,17]. This process is known as the gain-of-toxic (GOT) of TDP-43. TDP-43 aggregates are one of the key markers for verifying its pathogenicity. Pathological aggregates of TDP-43 are the canonical neuropathological hallmark of ALS, which has been definitively validated by extensive clinical and neuropathological studies worldwide [18,19,20]. The formation of aggregates is usually accompanied by hyperphosphorylation and ubiquitination of TDP-43 [21,22]. Pathological TDP-43 aggregates can further impair mitochondrial function, block the autophagy-lysosomal pathway, and accelerate motor neuron death [23,24,25,26]. Mutant TDP-43 protein shows enhanced cytoplasmic mislocalization [27]. Meanwhile, aberrant nucleocytoplasmic shuttling and aggregate formation lead to massive depletion of nuclear TDP-43 [28,29], rendering it unable to perform its physiological functions such as RNA regulation [30]. This mechanism is known as loss-of-function (LOF) of TDP-43 [31,32,33,34]. The mechanistic role of TDP-43 in ALS pathogenesis has not been fully elucidated, and it is widely accepted that the disease is driven by the synergistic effects of GOT and LOF [35,36].

In clinical practice, *TARDBP* variants of uncertain significance pose substantial challenges to the diagnosis and treatment of ALS. To date, more than 172 *TARDBP* variants have been reported, yet the pathogenicity has been definitively established for only approximately one-third of these variants. In this study, we systematically classified the pathogenicity of all 172 reported *TARDBP* variants cataloged in the Human Gene Mutation Database (HGMD) and ClinVar database up to the present, in strict accordance with the standards and guidelines issued by the ACMG. However, the pathogenicity of 104 variants remains unclarified. To address this gap, this study started from the canonical pathogenic mechanisms of TDP-43 to evaluate the functional impacts of the mutant gene products. Furthermore, this study summarized the clinical characteristics of ALS patients carrying *TARDBP* mutations from published studies, and assessed the feasibility of functional validation using skin fibroblasts derived from patients with *TARDBP* mutations.

## 2. Materials and Methods

### 2.1. Pathogenicity Classification of Variants and Summary of Clinical Characteristics

All variants across the 5 coding exons of the *TARDBP* gene were included in the analysis. We systematically classified the pathogenicity of all 172 *TARDBP* variant loci cataloged in the HGMD (version 2025.10) and ClinVar database (version 2025.10) in strict accordance with the ACMG guidelines (Supplementary Table S1). Variants were excluded based on the following criteria: (1) variants with definitive ACMG classification (via InterVar and Franklin): Pathogenic (P), Likely Pathogenic (LP), Likely Benign (LB), Benign (B); (2) variants located in intronic regions; (3) insertion, duplication, or deletion mutations. Finally, a total of 28 *TARDBP* missense VUS were identified for subsequent analysis (Table 1). Subsequently, we conducted a comprehensive literature search for all published cases reporting these 28 variants, and extracted the following information from each eligible study: DNA or amino acid alteration, year of publication, article title, number of affected pedigrees, geographic region, family history, sex, age at onset, disease duration, and site of onset (Table 2).

### 2.2. Plasmid

The *TARDBP* complementary DNA (cDNA) was constructed with the reference sequence NM_007375.4 as the template. The 1245 base pair (bp) open reading frame (ORF) of *TARDBP* was cloned into the pEGFP vector and pIRES2 plasmid, respectively, using the ClonExpress II One Step Cloning Kit (Vazyme Biotech Co., Ltd., Nanjing, China). Subsequently, different *TARDBP* variants were generated via homologous recombination technology, and the accuracy of their sequences was verified by Sanger sequencing.

### 2.3. Cell Culture

HEK293T cells were cultured in Dulbecco’s Modified Eagle’s Medium (DMEM, HyClone Laboratories, Logan, UT, USA) supplemented with 10% fetal bovine serum (FBS, Gibco, Thermo Fisher Scientific, Waltham, MA, USA) at 37 °C in a humidified 5% CO_2_ incubator. Primary fibroblast cell lines were established from 4 mm skin biopsy specimens obtained from ALS probands and one healthy control subject. Fibroblasts were cultured in DMEM (Gibco, Thermo Fisher Scientific, Waltham, MA, USA) supplemented with 10% FBS (Gibco, Thermo Fisher Scientific, Waltham, MA, USA) and 1% penicillin-streptomycin antibiotic mixture (Gibco, Thermo Fisher Scientific, Waltham, MA, USA) at 37 °C in a humidified 5% CO_2_ atmosphere. The HEK293T cells were donated by Bao-Rong Zhang from the Second Affiliated Hospital, Zhejiang University School of Medicine.

### 2.4. Immunofluorescence Staining

HEK293T cells or fibroblasts were seeded at a density of 0.8 × 10^4^ cells/mL into 24-well plates containing coverslips (WHB Biotechnology Co., Ltd., Shanghai, China) pre-coated with Poly-D-lysine hydrobromide (Sigma-Aldrich, Cat. No. P1149). HEK293T cells were transfected with the pIRES2 plasmid after 24 h of culture, while fibroblasts were not subjected to transfection. Transient transfection was performed using Lipofectamine 3000 reagent (Thermo Fisher Scientific, Waltham, MA, USA) following the manufacturer’s protocol. At 48 h post-transfection, the culture medium was discarded, and cells were washed with ice-cold phosphate-buffered saline (PBS). Cells were then fixed with 4% paraformaldehyde (PFA, Shanghai Institutes for Biological Sciences, Chinese Academy of Sciences, Shanghai, China) for 15 min at room temperature, followed by 3 washes with PBS. Cells were permeabilized with a 0.1% Triton X-100 solution for 10 min, and then blocked with 0.1% Triton X-100 containing 10% goat serum (GS) for 1 h at room temperature. Subsequently, cells were incubated with TDP-43 Polyclonal Antibody (1:400, Proteintech Group, Inc. Rosemont, IL, USA. Cat. No. 10782-2-AP.) overnight at 4 °C. On the next day, the primary antibody was removed, and the coverslips were washed 3 times with PBS, followed by incubation with fluorescent secondary antibody (1:10,000, Thermo Fisher Scientific, Cat. No. A-11012) for 1 h at room temperature, and another 3 washes with PBS. Nuclei were counterstained with Hoechst (COOLABER Science & Technology Co., Ltd. Beijing, China. Cat. No. SL7131) for 5 min at room temperature. After 3 additional washes with PBS, coverslips were mounted with anti-fade fluorescence mounting medium (SouthernBiotech, Birmingham, AL, USA. Cat. No. 0100-01). Cell imaging was performed using a Leica DM6B laser scanning confocal microscope. Images were acquired from random fields of view with a 63× oil immersion objective, and at least 3 fields were captured per sample. All experiments for each variant locus were repeated independently at least 3 times.

Aggregate-positive: cells contained either intranuclear aggregates with fluorescence intensity significantly higher than the nuclear background or non-diffuse cytoplasmic punctate aggregates. Aberrant nuclear translocation- positive: cells showing detectable TDP-43 protein expression in the extranuclear cytoplasmic region, with a fluorescence intensity not lower than the background nuclear TDP-43 signal intensity in cells without plasmid transfection.

### 2.5. Western Blotting

HEK293T cells were seeded at a density of 1 × 10^5^ cells/mL into 12-well plates. After 24 h of culture, cells were transfected with the corresponding pEGFP plasmids, respectively. Transient transfection was performed using Lipofectamine 3000 reagen in strict accordance with the manufacturer’s instructions. At 48 h post-transfection, HEK293T cells overexpressing TDP-43 protein were lysed and collected. Total protein concentration of cell lysates was quantified using a Bicinchoninic Acid (BCA) Protein Assay Kit (Takara Bio Inc., Kusatsu, Shiga, Japan. Cat. No. T9300A). Equal amounts of protein samples were separated by sodium dodecyl sulfate-polyacrylamide gel electrophoresis (SDS-PAGE), transferred onto polyvinylidene fluoride (PVDF) membranes, and blocked with 5% non-fat milk. Immunoblotting was performed by overnight incubation with primary antibodies at 4 °C. The primary antibodies used were as follows: Phospho-TDP43 (Ser409/410) Polyclonal Antibody (1:3000, Proteintech Group, Inc., Cat. No. 22309-1-AP), HRP-conjugated GAPDH Mouse Monoclonal Antibody (1:10,000, ABclonal Technology, Woburn, MA, USA. Cat. No. AC035), LC3A/B (D3U4C) Rabbit Monoclonal Antibody (Cell Signaling Technology, Danvers, MA, USA. Cat. No. #12741), and SQSTM1/p62 (D5L7G) Mouse Monoclonal Antibody (Cell Signaling Technology, CST, Cat. No. 88588). All immunoblot images were acquired using a Bio-Rad imaging system. Quantitative analysis of protein bands was performed using ImageJ software (version 1.52v, National Institutes of Health, Bethesda, MD, USA).

### 2.6. Genetic Analyses and Sanger Sequencing

We extracted genomic DNA from participants’ peripheral blood samples by using QIAamp blood genomic extraction kits (Qiagen, Hilden, Germany) following the standard protocols. PCR amplification was performed on the *TARDBP* gene of the proband. Sanger sequencing was carried out to validate the variant and co-segregation in each proband and available familial members.

### 2.7. Statistical Analyses

Data are presented in the figures as the mean ± standard deviation. Statistical analyses were performed using Student’s *t*-test in GraphPad Prism 9 software (La Jolla, CA, USA). Statistical significance was defined as *p* value < 0.05. Differences were considered statistically significant at * *p* < 0.05, ** *p* < 0.01, *** *p* < 0.001 or **** *p* < 0.0001. All statistical tests were preceded by normality tests and homogeneity of variance analyses. For samples with unequal variances, Welch’s *t*-test was applied. For samples that did not follow a normal distribution, the Mann–Whitney U test was used. The Bonferroni correction was applied to adjust the significance level for multiple comparisons.

## 3. Results

### 3.1. Pathogenicity Classification of TARDBP Variants of Uncertain Significance Based on Aggregate Formation and Nuclear Translocation Abnormalities

**Figure 2:**
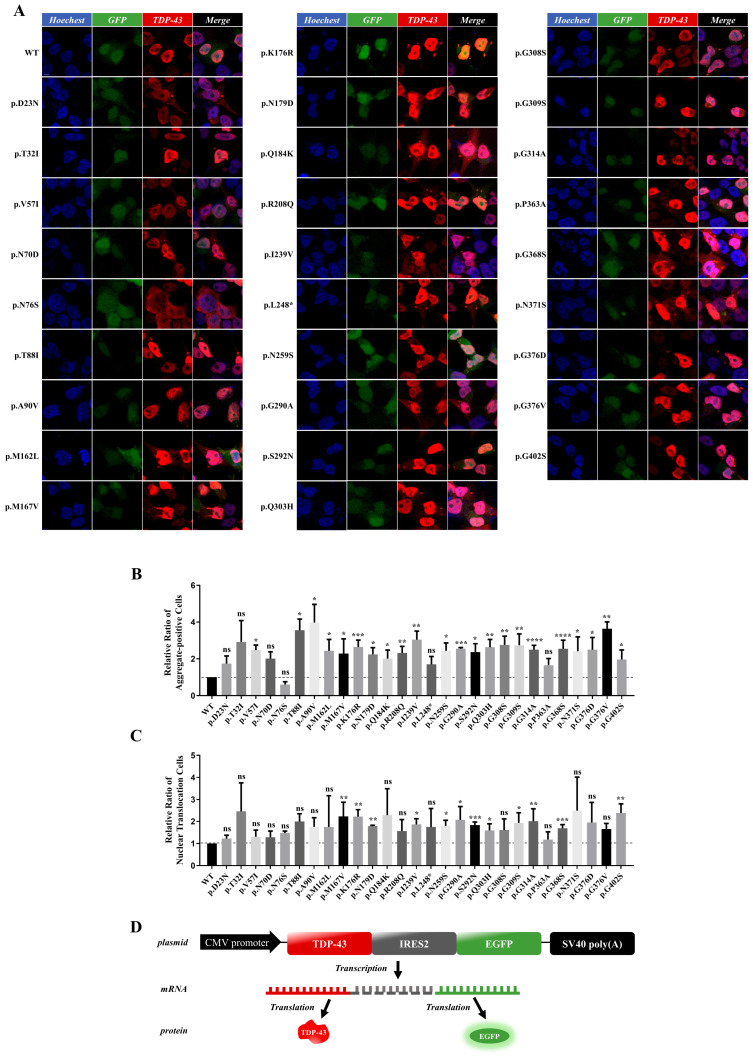
Effects of 28 *TARDBP* variants of uncertain significance on TDP-43 aggregate formation and nuclear translocation capacity. (**A**) HEK293T cells were transiently transfected with wild-type or mutant TDP-43 plasmids for 48 h and observed under confocal microscopy. Nuclei are visualized with blue fluorescence; EGFP (green fluorescence) indicates successful transfection; TDP-43 is labeled with red fluorescence. Scale bar: 5 μm. (**B**) Quantitative comparison of the percentage of aggregate-positive cells between the wild-type and mutant variants. (**C**) Quantitative comparison of the percentage of cells with aberrant nuclear translocation between the wild-type and mutant variants. (**D**) Schematic diagram of the expression cassette of the pIRES2 plasmid. Data are presented as mean ± standard deviation (SD). ns, not significant. * *p* < 0.05, ** *p* < 0.01, *** *p* < 0.001, **** *p* < 0.0001 vs. wild-type.

First, we analyzed the 28 screened *TARDBP* variant loci of VUS (Table 1). To further clarify their pathogenicity, we transiently transfected plasmids encoding wild-type (WT) or mutant TDP-43 into HEK293T cells [37]. We selected the pIRES2 plasmid as the expression vector for these mutant TDP-43 proteins. Leveraging its internal ribosomal entry site (IRES) element, the pIRES2 vector enables separate expression of TDP-43 and EGFP without generating a fusion protein (Figure 2D). This method enabled the exclusion of non-transfected cells during quantification and avoided false-positive results from spontaneous EGFP aggregation. We selected four well-established B or LB and four well-established P or LP *TARDBP* variants as controls (Figure S1). According to the recommendations from the ClinGen Sequence Variant Interpretation (SVI) Working Group [38], the strength of evidence from our study can be classified as PS3_ supporting. GOT and LOF of TDP-43 are two entirely distinct pathogenic mechanisms. Therefore, we regard the evidence for GOT and LOF as independent PS3_supporting evidence respectively. We categorize the combined evidence as PS3_moderate when both pieces of PS3_supporting evidence exist concurrently [38].

At 48 h post-transfection, we first quantified the number of EGFP-positive cells, and subsequently observed the morphology and localization of TDP-43 within these EGFP-positive cells. We first assessed the number of cells with TDP-43 aggregate formation. In the WT TDP-43 control group, TDP-43 aggregate formation was detected in only a small proportion of cells. Of note, a large number of aggregate-positive cells were clearly observed in cells transfected with 27 of the VUS (Figure 2A). Following statistical analysis, 22 variants still exhibited a statistically significant difference in aggregate formation compared with the WT (Figure 2B). Among them, the percentage of aggregate-positive cells for variants p.D23N, p.T32I, p.N70D and p.P363A showed an increasing trend but did not reach statistical significance; the percentage of aggregate-positive cells for the p.N76S variant appeared to decrease, which also did not reach statistical significance.

We subsequently further compared the aberrant cytoplasmic mislocalization of mutant TDP-43 (Figure 2C). We found that aberrant TDP-43 mislocalization was also prevalent across these variants. Although the percentage of cells with abnormal nuclear translocation was elevated in nearly half of the variants, this increase did not reach statistical significance. This may be attributed to the fact that the nucleocytoplasmic shuttling of TDP-43 is a dynamic process, leading to phenotypic variability.

Therefore, based on in silico pathogenicity prediction, population minor allele frequency (MAF), combined with robust functional experimental evidence, we classified 22 *TARDBP* VUS as functionally abnormal. According to the recommendations from the ClinGen SVI Working Group [38], 12 *TARDBP* VUS could be reclassified as LP (Table 1). We further indicated in Table 1 whether the evidence was derived from TDP-43 GOF or LOF.

### 3.2. Cellular Accumulation of Phosphorylated TDP-43 in TARDBP Mutants

**Figure 3:**
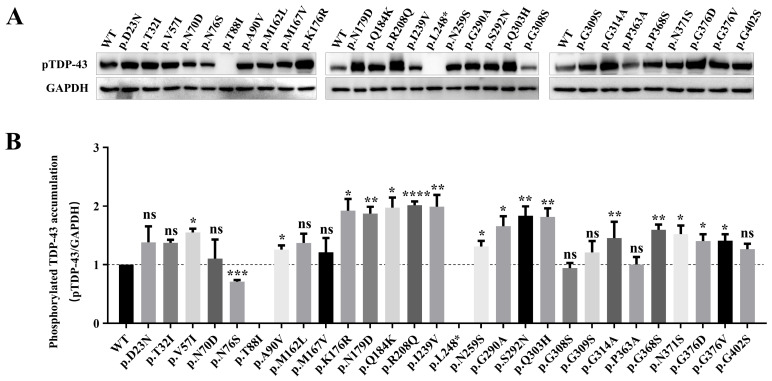
Analysis of phosphorylated TDP-43 protein cellular accumulation levels in wild-type and mutant variants. (**A**) HEK293T cells were transiently transfected with pEGFP-TDP-43 vectors encoding wild-type or mutant TDP-43, followed by extraction of total cellular protein and immunoblot analysis. (**B**) Values represent mean ± SD, n = 3. ns, not significant. * *p* < 0.05, ** *p* < 0.01, *** *p* < 0.001, **** *p* < 0.0001 vs. wild-type.

To further investigate the relationship between different *TARDBP* variants and TDP-43 phosphorylation (Ser409/410), we assessed the cellular accumulation levels of mutant TDP-43 (Figure 3A,B). At 48 h post-transfection with the pEGFP plasmids, TDP-43 fusion proteins extracted from HEK293T cells were detected by Western blotting. The results showed that 16 variants (p.V57I, p.A90V, p.K176R, p.N179D, p.Q184K, p.R208Q, p.I239V, p.N259S, p.G290A, p.S292N, p.Q303H, p.G314A, p.G368S, p.N371S, p.G376D, p.G376V) exhibited significantly elevated levels of pTDP-43 accumulation compared with WT TDP-43. Furthermore, the pTDP-43 accumulation levels of the p.D23N, p.T32I and p.G402S variants were moderately increased, but the difference did not reach statistical significance.

Of note, the p.N76S variant showed reduced pTDP-43 accumulation levels relative to the WT. Interestingly, the protein band of TDP-43 carrying the p.T88I variant was located above the band of WT TDP-43, at approximately 120 kDa. The p.L248* variant is a truncating mutation, with its protein band detected at approximately 55 kDa (Figure S2). Therefore, we did not compare the pTDP-43 accumulation levels of these two variants.

### 3.3. Clinical Characteristics and Genotype-Phenotype Correlation Analysis of ALS Patients Carrying 12 TARDBP Variants

We performed a systematic literature review of all 28 identified VUS in the *TARDBP* gene. A total of 35 patients (including 1 previously unpublished patient from our center) carried 12 *TARDBP* VUS variants, and their clinical and demographic characteristics are summarized in Table 2. After excluding cases with missing clinical data, 74.3% (26/35) of patients were diagnosed with familial amyotrophic lateral sclerosis (fALS), and the remaining 25.7% (9/35) were diagnosed with sporadic amyotrophic lateral sclerosis (sALS). The male-to-female ratio was 1:0.83, with a slight male predominance.

**Figure 4:**
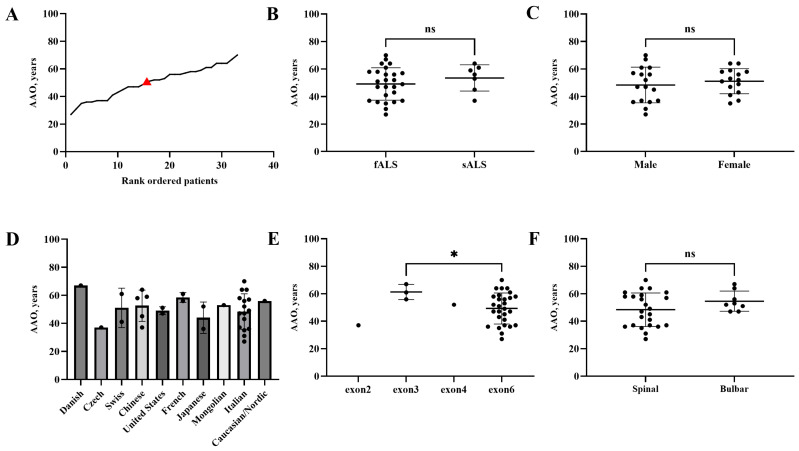
Age at onset of patients carrying *TARDBP* mutations. (**A**) Plot of rank ordered *TARDBP*-mutant patients showing the mean AAO of 50.1 years (the point marked by the red triangle). (**B**) Plots comparing fALS patients with sALS patients with no significant difference in AAO. (**C**) Plots comparing male and female patients with no significant difference in AAO. (**D**) Plots comparing the AAO among patients carrying mutations in different countries. (**E**) Plots comparing the AAO among patients carrying mutations in different exons of the *TARDBP* gene. The AAO of patients carrying mutations in exon 6 was earlier than those in exon 3 (*p* = 0.0332). (**F**) Plots comparing spinal onset and bulbar onset patients with no significant difference in AAO. ns, not significant. * *p* < 0.05.

**Figure 5:**
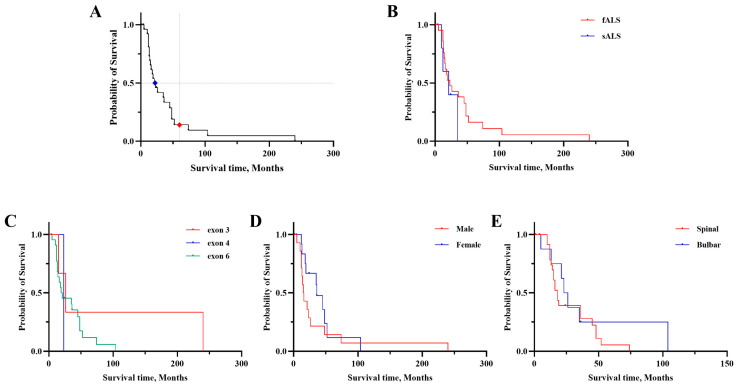
Survival analysis comparison of patients carrying *TARDBP* mutations. (**A**) Plot of survival probabilities for all subjects with *TARDBP* mutations. The overall median survival time was seen at blue mark (22 months) and the 5 year survival rate was seen at red (14.4%). (**B**) Plots of survival probabilities between all fALS and sALS patients (median survival 23 months vs. 21 months). (**C**) Plots of survival probabilities among patients with different exons in *TARDBP* (median survival in exon3 vs. exon4 vs. exon6, 26 months vs. 23 months vs. 20 months). (**D**) Plots of survival probabilities between all male and female patients (median survival 15.5 months vs. 36 months). (**E**) Plots of survival probabilities between spinal onset and bulbar onset patients (median survival 18 months vs. 24.5 months).

Among the 35 patients with available clinical data, 1 case with missing onset site data was excluded. Of the remaining 34 patients, 25 (73.5%) presented with spinal (limb) onset, 8 (23.5%) with bulbar onset, and 1 (2.9%) with psychiatric symptoms dominated by personality changes as the initial manifestation. The distribution of age at onset (AAO) of patients carrying *TARDBP* variants is shown in Figure 4A, with a mean AAO (standard deviation, SD) of 50.1 ± 11.3 years. The mean AAO of fALS (49.2 ± 11.7 years) was earlier than that of sALS patients (53.6 ± 9.6 years), but the difference did not reach statistical significance (Figure 4B). Similarly, no statistically significant differences in AAO or overall survival were observed among groups stratified by sex, geographical region, or site of onset (Figure 4C,D,F). Of note, ALS patients harboring pathogenic *TARDBP* variants in exon 6 had a significantly younger mean AAO (49.3 ± 11.3 years) compared with those carrying variants in *TARDBP* exon 3 (61.3 ± 5.5 years). The overall median overall survival (mOS) of patients carrying *TARDBP* variants was 22 months, with a 5-year overall survival rate of 14.4% (Figure 5A). No statistically significant differences in survival were observed across all analyzed clinical subgroups, including fALS versus sALS, patients with *TARDBP* variants in different exons, male versus female patients, and spinal-onset versus bulbar-onset ALS patients (Figure 5B–E).

**Figure 6:**
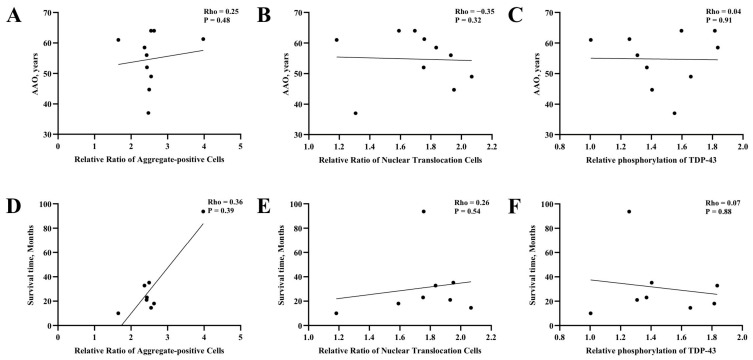
Correlation analysis of mutated TDP-43 cellular functional phenotypes with clinical phenotypes. (**A**) Correlation between the relative ratio of aggregate-positive cells and the AAO among all subjects (rho = 0.25, *p* = 0.48). (**B**) Correlation between the relative ratio of nuclear translocation cells and the AAO among all subjects (rho = −0.35, *p* = 0.32). (**C**) Correlation between the relative phosphorylation of TDP-43 and the AAO among all subjects (rho = 0.04, *p* = 0.91). (**D**) Correlation between the relative ratio of aggregate-positive cells and the mean survival time among all subjects (rho = 0.36, *p* = 0.39). (**E**) Correlation between the relative ratio of nuclear translocation cells and the mean survival time among all subjects (rho = 0.26, *p* = 0.54). (**F**) Correlation between the relative phosphorylation of TDP-43 and the mean survival time among all subjects (rho = 0.07, *p* = 0.88).

In addition, we analyzed the correlation between cellular functional phenotypes of TDP-43 (aggregate formation level, aberrant nuclear translocation level, and phosphorylation level) and clinical phenotypes in this study. However, no significant correlation was observed between these cellular phenotypes and AAO or overall survival duration (Figure 6).

We noted that incomplete penetrance is a highly prevalent feature of *TARDBP* variants. The p.S292N variant was identified in the proband and 4 asymptomatic relatives [39]. One ALS patient carried both the p.A90V and p.G357R variants, while one of his family members in his 70s, who also carried both variants, remained asymptomatic [40].

### 3.4. Functional Validation of the Novel TARDBP Variant p.A382S

**Figure 7:**
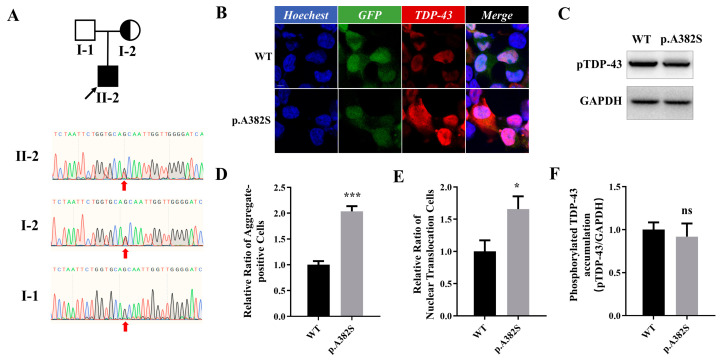
Validation of the pathogenicity of the new mutation p.A382S. (**A**) Pedigree and Sanger verification of *TARDBP* p.A382S. (**B**) HEK293T cells were transiently transfected with wild-type or p.A382S TDP-43 plasmids for 48 h and observed under confocal microscopy. Nuclei are visualized with blue fluorescence; EGFP (green fluorescence) indicates successful transfection; TDP-43 is labeled with red fluorescence. Scale bar: 5 μm. (**C**) HEK293T cells were transiently transfected with pEGFP-TDP-43 vectors encoding wild-type or p.A382S TDP-43, followed by extraction of total cellular protein and immunoblot analysis. (**D**) Quantitative comparison of the percentage of aggregate-positive cells between the wild-type and mutant variants. (**E**) Quantitative comparison of the percentage of cells with aberrant nuclear translocation between the wild-type and mutant variants. (**F**) Quantitative analysis of phosphorylated TDP-43 protein levels. Data are presented as mean ± standard deviation (SD). ns, not significant. * *p* < 0.05, *** *p* < 0.001 vs. wild-type.

The same incomplete penetrance was also observed in our center: we identified a novel unreported *TARDBP* variant in a 38-year-old male patient with left lower limb onset. Whole-exome sequencing detected the *TARDBP* variant: c.1144G>T (p.A382S). The Ala residue at position 382 of TDP-43 is a well-established mutation hotspot, and the pathogenicity of variants at this site (such as p.A382T and p.A382P) has been fully validated [41,42]. Pathogenicity prediction classified the p.A382S variant as Likely Pathogenic (LP). This variant was inherited from the patient’s mother (Figure 7A). At the time of the patient’s clinical assessment, the mother was 63 years of age and had not developed any clinical symptoms of ALS or other related neurodegenerative disorders.

Given that p.A382S has not been previously reported and exhibits incomplete penetrance, we performed the same panel of functional assays described above to further characterize the functional consequences of this novel variant. The results showed that p.A382S presented with pronounced aggregate formation and aberrant nuclear translocation (Figure 7B,D,E). We subsequently detected the phosphorylation level of TDP-43 carrying this variant. Unexpectedly, p.A382S did not exhibit elevated phosphorylation level (Figure 7C,F). These findings suggest that different substitutions affecting the same amino acid residue may be associated with distinct functional consequences and potentially different pathogenic mechanisms.

We further detected the expression of core autophagy markers in cells carrying the p.A382S variant (Figure S3). The LC3B/LC3A ratio was significantly increased, and p62 protein level showed an upward trend without statistical significance. These preliminary findings suggest alterations in autophagy-related markers that may be associated with the p.A382S variant. However, the pathogenicity and underlying mechanism of p.A382S require further investigation.

### 3.5. Establishing Variant Pathogenicity via Fibroblast TDP-43 Aggregation

**Figure 8:**
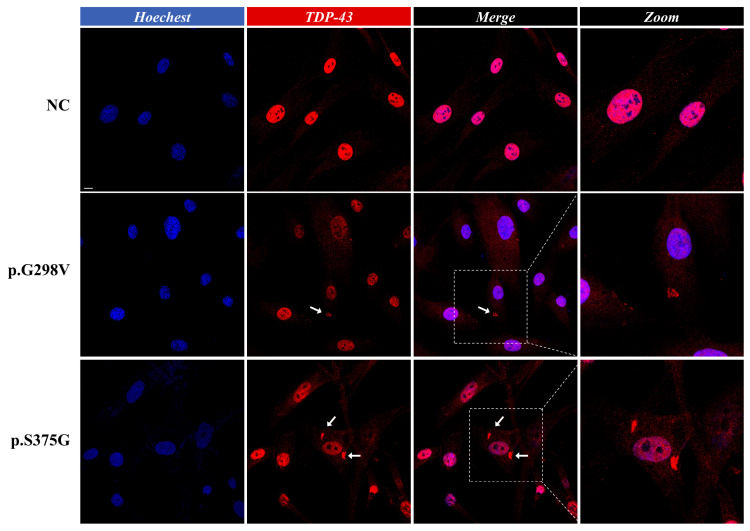
Mutant TDP-43 aggregates formation by immunofluorescence. Immunofluorescence staining of fibroblasts carrying *TARDBP* p.G298V or p.S375G variants and control fibroblasts. Cells were labeled with an antibody against TDP-43 (red). Scale bars: 10 um. White arrows indicate TDP-43 aggregates.

The marked clinical heterogeneity and incomplete penetrance of pathogenic *TARDBP* variants pose substantial challenges to the clinical diagnosis and genetic counseling of ALS. We sought to identify a simple and robust method for the pathogenicity validation of *TARDBP* variants [43]. We isolated, cultured, and performed IF staining on skin fibroblasts derived from ALS patients carrying *TARDBP* mutations. Observation via laser scanning confocal microscopy revealed that a subset of cells presented with prominent TDP-43 protein-positive aggregates in the cytoplasm, which appeared as irregular clumpy accumulations (Figure 8). In skin fibroblasts from healthy controls, TDP-43 protein exhibits a diffuse and homogeneous nuclear distribution, with no cytoplasmic mislocalization or pathological aggregate formation. This method provides a feasible strategy for the pathogenicity classification of VUS in clinical practice.

Emerging evidence has linked translational stress, dysregulated RNA-binding protein homeostasis, and aberrant stress granules (SGs) persistence to impaired proteostasis in neurodegenerative diseases including ALS and frontotemporal dementia (FTD) [44,45]. As a core scaffold protein of SGs, Ras GTPase-activating protein-binding protein 1 (G3BP1) is closely coupled with the pathological aggregation of TDP-43 [46,47]. We further assessed the expression pattern and localization of G3BP1 in patient-derived skin fibroblasts (Figure S4). No significant difference in G3BP1 expression or baseline SG assembly was observed between the *TARDBP* mutant group and the healthy control group. Given that persistent or abnormal SGs may promote aberrant aggregation of TDP-43 in the cytoplasm [44], this mechanism merits further investigation. Notably, our current analysis was restricted to basal G3BP1 localization under steady-state conditions and did not directly examine the kinetics of SG assembly, persistence, or clearance upon stress stimulation; thus, these results should be interpreted with caution, and the potential role of dysregulated SG dynamics in TDP-43 pathogenesis warrants further exploration in dedicated functional studies.

## 4. Discussion

In this study, we evaluated the pathogenicity of 28 *TARDBP* VUS via functional assays. Of these, 22 variants were classified as functionally abnormal, and 12 *TARDBP* VUS could be reclassified as LP (Table 1). *TARDBP* variants were distributed across exons 2 to 6, with the majority localized to the CTD. In our study, all variants that could be reclassified as LP are located in exon 6. Subsequently, we summarized the clinical characteristics of ALS patients carrying the 28 VUS variants. This is the first study to date that systematically analyzes the pathogenicity of all reported *TARDBP* VUS. Our findings provide a basis for clinicians to achieve earlier diagnosis of ALS, and facilitate more accurate interpretation of the clinical phenotypes of patients carrying *TARDBP* mutations.

The pathogenic mechanisms underlying TDP-43-related ALS have not yet been fully elucidated. In this study, we assessed aggregate formation of mutant TDP-43 as the core functional readout for GOT, and evaluated aberrant nuclear translocation of TDP-43 as a key surrogate marker for LOF, to clarify the pathogenicity of these variants. The core functional domains of TDP-43 consist of the NTD, RRM1/2, and CTD, with the majority of pathogenic mutations located in the CTD region [48]. Mutations in different functional domains may exert pathogenic effects via distinct mechanisms [49]. For instance, p.I239V and p.L248* are situated in the NES region. These variants may drive aggregate formation by impairing the physiological nucleocytoplasmic transport function of TDP-43. The CTD of TDP-43 is the core functional domain that mediates protein phase separation and pathological aggregation, and its sequence is inherently highly enriched in glycine and glutamine residues [50]. Pathogenic mutations localized to this region might enhance the intrinsic aggregation propensity of TDP-43 and accelerate the formation of its pathological aggregates. The role of the NTD in the pathogenic mechanism of mutant TDP-43 has received increasing attention, as dimerization of this domain plays a critical role in regulating its RNA splicing activity and the formation of pathological TDP-43 inclusions [51]. p.T88I variant, may enhance NTD-mediated dimerization by disrupting the conformational integrity of the NTD. This mechanistically explains, at least in part, the higher apparent molecular weight of TDP-43 carrying the p.T88I variant compared with WT TDP-43.

In recent years, the LOF of TDP-43 has received extensive attention [27,52,53,54,55]. Previous studies have confirmed that TDP-43 maintains the expression of Stathmin 2 (*STMN2*) via regulating its pre-mRNA splicing [56]. The *STMN2*-encoded Stathmin 2 protein is a key regulator of microtubule dynamics and homeostasis in neuronal axons. Pathogenic TDP-43 mutants suppress the production of full-length functional Stathmin 2 via inducing aberrant *STMN2* splicing, which disrupts axonal cytoskeletal integrity and axonal electrical conduction, ultimately leading to motor neuron injury and apoptotic death [57]. In this study, we found that many of the identified TDP-43 mutants were accompanied by significant aberrant nucleocytoplasmic localization. Notably, the nuclear mislocalization observed in this study is only characterized as “functional abnormal” (Table 1), rather than directly classified as evidence meeting PVS1 or PS3. In this work, the aberrant cytoplasmic localization of TDP-43 serves solely as indirect evidence of impaired nuclear function. We did not further investigate the transcriptomic alterations of downstream target RNAs or the disruption of protein–protein interaction networks mediated by these mutants, which constitutes a limitation of the current study. To date, no conclusive evidence supports that TDP-43 LOF alone is sufficient to drive ALS pathogenesis. Accordingly, we only treat nuclear mislocalization as supplementary functional abnormal evidence to upgrade the evidence strength from PS3_supporting to PS3_moderate, and this adjustment is only valid when PS3_supporting evidence derived from GOT is confirmed. When nuclear mislocalization is the sole observed phenotype, it does not qualify for PS3 or PVS1 evidence levels. Despite this, our findings still provide novel experimental evidence supporting that TDP-43 LOF is an important independent pathogenic mechanism.

Hyperphosphorylated TDP-43 is a canonical pathological hallmark of ALS [19,22]. In post-mortem brain tissues of ALS patients, hyperphosphorylated TDP-43 aggregates can be observed [22,31,58]. This hyperphosphorylation was traditionally considered a key step in the conversion of TDP-43 from a soluble nuclear protein to insoluble cytoplasmic inclusions [58,59]. However, recent studies have suggested that TDP-43 hyperphosphorylation may not be a sole pathogenic driver, but rather a self-protective mechanism initiated by cells [60,61,62,63]. In this study, we found that not all variants that induced TDP-43 aggregate formation exhibited a significant elevation in phosphorylation levels, such as p.V57I. In contrast, reduced aggregate formation appeared to be accompanied by decreased phosphorylation levels, as exemplified by the p.N76S variant. These findings suggest that phosphorylation is one of the key regulators of TDP-43 aggregate formation. Of note, the p.A382S variant was functionally characterized for the first time in this study. The Ala residue at position 382 of TDP-43 is a well-established mutation hotspot, and previous studies have reported that the p.A382T variant drives TDP-43 aggregate formation and hyperphosphorylation [64]. However, distinct from p.A382T, p.A382S presented with TDP-43 aggregation and aberrant mislocalization, but no increase in pTDP-43 accumulation levels.

The mean AAO of ALS patients carrying *TARDBP* mutations was 50.1 years, and 73.5% of patients presented with limb-onset disease. No statistically significant differences were observed in age at onset, disease progression, or clinical phenotypes between groups stratified by sex or geographical region. Of note, patients carrying mutations in exon 6 had a significantly earlier age at onset than those with variants in exon 3, suggesting that variants localized to the CTD may confer higher pathogenicity.

*TARDBP* mutations are associated with marked clinical heterogeneity in ALS. The p.G376D variant has been previously reported in an Italian pedigree [65], in which 12 family members were diagnosed with ALS. The AAO ranged from as early as 27 years to as late as 70 years in this pedigree. Most patients presented with spinal onset, predominantly involving the upper limbs, but 1 case had bulbar onset. The overall disease progression was extremely rapid, with a median disease duration of only 11 months, and most patients required early tracheotomy, but 1 patient with late onset at 70 years had a relatively slow disease course. The p.G376D variant exhibited significant intrafamilial heterogeneity in AAO and disease progression. The p.S292N variant has been reported in a Chinese pedigree [66], where the proband was a 64-year-old female with bulbar onset; in another independent sporadic ALS patient carrying the p.S292N variant [39], the disease presented with upper limb onset. One patient carrying the *TARDBP* p.A90V variant presented with behavioral variant frontotemporal dementia (bvFTD) at the age of 56 years, with core manifestations of personality changes, compulsive behavior, food hoarding, and social disinhibition [67]. Motor symptoms, including fasciculation and proximal muscle weakness, gradually appeared 11 years after disease onset, and ALS was confirmed 20 years after initial presentation, with an overall disease duration of more than 20 years, which was significantly slower than the progression of classic TDP-43-related neurodegenerative diseases.

Different amino acid substitutions at the same residue of TDP-43 can also lead to distinct clinical phenotypes and pathogenic mechanisms. For instance, substitution of the Gly residue at position 376 of TDP-43 to Asp results in a typical ALS phenotype. In contrast, the p.G376V variant from a French pedigree presented with progressive distal muscle weakness and atrophy, and was finally diagnosed as late-onset distal myopathy rather than ALS. Although both the p.A382S and p.A382T variants present with an ALS phenotype [68], they exhibited different patterns of pTDP-43 accumulation, which may reflect differences in their underlying biological effects. Notably, in published large-scale genetic screening cohorts of Italian ALS patients, the p.A382T variant is the most prevalent pathogenic mutation in the *TARDBP* gene, with well-documented extreme clinical phenotypic heterogeneity [65]. Approximately 30% of p.A382T-carrying ALS patients develop concurrent cognitive impairment. More strikingly, the patient harboring the p.A382P variant exhibited an atypical clinical phenotype: severe sensory impairment occurred first as the presenting feature, followed by the onset of motor deficits, consistent with a slowly progressive mixed sensorimotor neuronopathy [69].

The incomplete penetrance of *TARDBP* variants also warrants attention. In this study, the p.S292N, p.G376D, and p.A382S variants were all classified as LP, yet asymptomatic carrier relatives were identified for each variant, which warrants careful clinical evaluation and genetic counseling in practice. In the Italian ALS patient cohort mentioned in the text [65], most of the pathogenic alleles in affected patients are inherited from unaffected healthy carriers, further supporting the incomplete penetrance of *TARDBP* variants highlighted in our study.

p.P363A has been previously reported in a French sporadic ALS patient. However, no familial co-segregation analysis was available for this pedigree, so we could neither confirm that this is a de novo *TARDBP* mutation, nor rule out the possibility that it was inherited from an asymptomatic parent with incomplete penetrance. The patient presented with lower limb-onset disease with typical ALS features. However, in our in vitro cellular functional assays, p.P363A did not exhibit significant pathogenicity: there was no increase in aggregate formation, aberrant nuclear translocation, or phosphorylation levels of TDP-43. We hypothesize that this variant may exert its pathogenic effects through alternative mechanisms, such as disrupting mitochondrial function or impairing the function of downstream proteins. The unique characteristics of this variant warrant further in-depth investigation. Therefore, further familial studies incorporating co-segregation analysis and other functional assays are urgently warranted to validate the pathogenicity of this variant. Such variants may represent incidentally identified benign germline variants, or context-dependent pathogenic mutations restricted to specific pedigrees, which remain to be further elucidated.

We also collected skin biopsy samples, performed primary culture and specific IF staining on skin fibroblasts derived from ALS patients carrying *TARDBP* gene mutations, and observed the presence of abnormal TDP-43 protein aggregates in these cells. This included samples from patients carrying the p.G298V and p.S375G variants, which have been previously definitively classified as LP. These results suggest that the pathological TDP-43 aggregation in skin fibroblasts can reflect the disease-related protein misfolding and aggregation characteristics in the central nervous system to some extent, and provide experimental evidence in the peripheral tissue for elucidating the pathological mechanisms of ALS. Meanwhile, given that skin biopsy is minimally invasive and fibroblasts are easily accessible, the detection method for TDP-43 aggregates holds potential clinical application value [70].

According to the recommendations of functional evidence from the ClinGen SVI Working Group [38], PS3 evidence is graded into four levels (supporting, moderate, strong, very strong) based on assay validation rigor. Its strength depends on the total number of independently classified pathogenic/benign controls and the assay’s discriminative performance. Insufficient controls for rare diseases pose a core challenge for functional validation of the ALS-associated *TARDBP* gene. Public databases only catalog 8 B or LB *TARDBP* variants, 4 of which are intronic. Our protein functional assay cannot evaluate intronic splicing effects, leaving only 4 valid exonic benign controls. More robust models may emerge with further TDP-43 mechanism research and control accumulation. Nevertheless, this study demonstrated that 22 out of 28 *TARDBP* VUS exhibited functionally abnormal results in this assay. By integrating additional other evidence, we ultimately upgraded 12 of these VUS to LP variants.

Several limitations of the present study should be acknowledged. First, the functional assays were primarily performed in HEK293T cells using transient overexpression systems. This platform provides a standardized and reproducible approach for the comparative functional assessment of a large number of *TARDBP* variants. However, it was not designed to comprehensively model neuronal disease biology and therefore cannot fully recapitulate the cellular and molecular complexity of ALS. Future studies using patient-derived iPSC motor neurons and other physiologically relevant neuronal models will be necessary to validate and extend these findings. In addition, total exogenous TDP-43 expression was not systematically quantified across all constructs. Therefore, the relative contributions of expression differences and intrinsic aggregation propensity to the observed phenotypes could not be fully distinguished. Second, the clinical and fibroblast analyses were limited by the relatively small number of available *TARDBP* mutation carriers. Several variants were represented by only a single patient, which may limit the statistical power of genotype–phenotype correlations, age-at-onset analyses, and survival comparisons. Accordingly, these results should be interpreted with caution and regarded strictly as exploratory. In addition, the fibroblast experiments included only two patients carrying likely pathogenic *TARDBP* variants and one healthy control, which should be regarded as preliminary, and larger independent cohorts will be required to evaluate the reproducibility and diagnostic performance of fibroblast-based functional assays. Finally, this study focused exclusively on missense *TARDBP* variants currently classified as VUS. Although these variants represent the largest category requiring clinical interpretation, our findings may not be directly applicable to other classes of *TARDBP* variants, including nonsense, frameshift, splice-site, or structural variants, which may act through distinct pathogenic mechanisms. In addition, not all reported *TARDBP* variants were functionally evaluated in the present study. Therefore, the conclusions should be interpreted within the context of missense VUS classification rather than the entire *TARDBP* mutational spectrum.

## 5. Conclusions

In summary, we systematically assessed the pathogenicity of 28 *TARDBP* VUS using a panel of functional assays. Our results demonstrated that 25 variants displayed significant functional defects, and 13 of these VUS could be upgraded to LP based on the functional evidence. We found that the majority of *TARDBP* pathogenic missense variants are clustered in the CTD of the encoded TDP-43 protein, and variants proximal to the C-terminus may lead to an earlier disease onset. The survival duration, site of onset, and clinical phenotypes of ALS patients carrying *TARDBP* variants are highly variable. Although missense variants only cause minimal changes at the genetic coding level, they confer significant phenotypic heterogeneity across patients. Given the incomplete penetrance of *TARDBP* variants, we recommend that individuals with an identified *TARDBP* gene variant, regardless of family history or clinical symptoms, should complete familial co-segregation verification and be evaluated for the risk of ALS. With the ongoing clinical trials of antisense oligonucleotide (ASO) therapy for ALS [71], our study further expands the mutation spectrum of pathogenic *TARDBP* variants. We anticipate that more studies on the pathogenicity classification of *TARDBP* variants will be conducted in the future.

## Figures and Tables

**Table 1:** Features and pathogenicity classification of 28 VUS variants in *TARDBP*.

Variant	Exon	Population Frequencies			Pathogenicity Predictions				ACMG Evidence	Functional Results	Additional Evidence	Final Classification
		gnomAD	1000G	ExAC	Revel	Alpha Missense	SIFT	MT				
c.67G>A (p.D23N)	2	.	.	9.0 × 10^−6^	U	U	U	D	PM2 + PP2	N	.	VUS
c.95C>T (p.T32I)	2	9.0 × 10^−6^	.	8.0 × 10^−6^	U	D	U	D	PM2 + PP2	N	.	VUS
c.169G>A (p.V57I)	2	.	.	.	U	B	B	D	PM2 + PP2	A (G)	PS3_supporting	VUS
c.208A>G (p.N70D)	2	.	.	.	B	B	B	D	PM2 + PP2	N	.	VUS
c.227A>G (p.N76S)	2	6.2 × 10^−5^	.	1.6 × 10^−5^	B	U	B	D	PM2 + PP2	N	.	VUS
c.263C>T (p.T88I)	3	.	.	.	B	B	B	D	PM2 + PP2 + BP4	A (G)	PS3_supporting	VUS
c.269C>T (p.A90V)	3	4.4 × 10^−4^	0	2.1 × 10^−4^	B	B	B	D	PM2 + PP2 + BP6	A (G)	PS3_supporting	VUS
c.484A>C (p.M162L)	4	.	.	.	B	B	B	D	PM2 + PP2 + BP4	A (G)	PS3_supporting	VUS
c.499A>G (p.M167V)	4	.	.	.	U	U	B	D	PM2 + PP2	A (G + L)	PS3_moderate	VUS
c.527A>G (p.K176R)	4	.	.	.	U	B	B	D	PM2 + PP2	A (G + L)	PS3_moderate	VUS
c.535A>G (p.N179D)	4	.	.	.	U	D	B	D	PM2 + PP2	A (G + L)	PS3_moderate	VUS
c.550C>A (p.Q184K)	5	.	.	.	B	B	B	B	PM2 + PP2	A (G)	PS3_supporting	VUS
c.623G>A (p.R208Q)	5	4.6 × 10^−5^	.	.	U	U	B	D	PM2 + PP2	A (G)	PS3_supporting	VUS
c.715A>G (p.I239V)	6	.	.	.	B	B	B	D	PM2 + PP2 + BP4	A (G + L)	PS3_moderate	VUS
c.743T>A (p.L248*)	6	.	.	.	.	.	.	D	PVS1 ^#^ + PM2	N	.	VUS
c.776A>G (p.N259S)	6	9.0 × 10^−6^	.	.	B	B	B	D	PM1 + PM2 + PP2	A (G + L)	PS3_moderate	LP
c.869G>C (p.G290A)	6	.	.	.	U	B	B	D	PM1 + PM2 + PP2	A (G + L)	PS3_moderate	LP
c.875G>A (p.S292N)	6	.	.	.	B	B	B	D	PM1 + PM2 + PP2	A (G + L)	PS3_moderate	LP
c.909A>C (p.Q303H)	6	1.8 × 10^−5^	.	.	U	B	B	D	PM1 + PM2 + PP2	A (G + L)	PS3_moderate	LP
c.922G>A (p.G308S)	6	.	.	.	B	B	B	D	PM1 + PM2 + PP2	A (G)	PS3_supporting	LP
c.925G>A (p.G309S)	6	3.1 × 10^−5^	.	.	U	B	U	D	PM1 + PM2 + PP2	A (G + L)	PS3_moderate	LP
c.941G>C (p.G314A)	6	.	.	.	U	U	B	D	PM1 + PM2 + PP2	A (G + L)	PS3_moderate	LP
c.1087C>G (p.P363A)	6	.	.	.	U	B	B	D	PM1 + PM2 + PP2	N	.	VUS
c.1102G>A (p.G368S)	6	1.7 × 10^−4^	.	2.6 × 10^−5^	U	B	B	D	PM1 + PM2 + PP2	A (G + L)	PS3_moderate	LP
c.1112A>G (p.N371S)	6	.	.	.	B	B	B	D	PM1 + PM2 + PP2	A (G)	PS3_supporting	LP
c.1127G>A (p.G376D)	6	.	.	.	U	U	U	D	PM1 + PM2 + PP2	A (G)	PS3_supporting	LP
c.1127G>T (p.G376V)	6	.	.	.	U	B	U	D	PM1 + PM2 + PP2	A (G)	PS3_supporting	LP
c.1204G>A (p.G402S)	6	.	.	.	U	B	U	D	PM1 + PM2 + PP2	A (G + L)	PS3_moderate	LP

Abbreviations: gnomAD = Genome Aggregation Database; 1000G = 1000 Genomes Project; ExAC = Exome Aggregation Consortium; Revel = Rare Exome Variant Ensemble Learner; SIFT = Sorting Intolerant From Tolerant; MT = MutationTaster. Revel results as Deleterious (D), Uncertain/Unknown (U) or Benign (B). Alpha Missense results as Damaging (D), Uncertain/Ambiguous (U) or Benign (B). SIFT results as Deleterious (D), Uncertain/Unknown (U) or Tolerated (T). MT results as Disease causing (D), Uncertain/Unknown (U) or Benign (B). VUS = Variant of Uncertain Significance; LP = Likely Pathogenic. G = Gain-of-toxicity; L = Loss-of-function. A = abnormal; N = normal. ^#^ PVS1_moderate.

**Table 2:** The demographic and clinical characteristics of the patients carrying *TARDBP* variants in this study.

Exon	Variants	Geographical Area	Case Count (n)	Male/Female	fALS/sALS	Bulbar Onset (%)	AAO, Years, Mean (SD)	Onset Range, Years	Mean Survival Time, Months, Mean (SD)
2	c.169G>A(p.V57I)	Czech Republic	1	0/1	0/1	0	37	37	.
3	c.269C>T(p.A90V)	Multiple countries	3	3/0	3/0	33.3	61.3 (5.5)	56–67	93.7 (126.8)
4	c.484A>C(p.M162L)	Italy	1	1/0	1/0	100	52	52	23
4	c.535A>G(p.N179D)	.	1	1/0	0/1	.	.	.	.
6	c.776A>G(p.N259S)	France	1	1/0	0/1	100	56	56	21
6	c.869G>C(p.G290A)	United States	2	1/1	2/0	50	49 (2.8)	47–51	14.5 (2.1)
6	c.875G>A(p.S292N)	China	4	0/4	2/2	50	58.5 (4.5)	53–64	>32.75 (5.9)
6	c.909A>C(p.Q303H)	Italy	1	0/1	1/0	0	64	64	18
6	c.1087C>G(p.P363A)	France	1	1/0	0/1	0	61	61	10
6	c.1102G>A(p.G368S)	Italy	1	.	0/1	0	64	64	.
6	c.1112A>G(p.N371S)	China	1	.	0/1	0	.	.	.
6	c.1127G>A(p.G376D)	Multiple countries	18	10/8	17/1	11.1	44.7 (11.0)	27–70	>35.2 (29.8)

Abbreviations: ALS = amyotrophic lateral sclerosis; fALS = familial ALS; sALS = sporadic ALS; AAO = age at onset.

## Data Availability

Part of the original contributions supporting the findings of this study are provided in the Supplementary Material. Further inquiries can be directed to the corresponding authors.

## Supplementary Materials

The following supporting information can be downloaded at: https://www.mdpi.com/article/10.3390/cells15141232/s1, Table S1: ACMG-Classified *TARDBP* Variants with Known Pathogenicity; Figure S1: Control assays with variants of established pathogenic classification; Figure S2: Expression of TDP-43 Protein Carrying p.T88I and p.L248* Variants; Figure S3: Expression Levels of Autophagy Markers LC3 and p62/SQSTM1 in Cells Expressing TDP-43 Carrying the p.A382S Varian; Figure S4: G3BP1 Expression in Skin Fibroblasts.
