# Supplementary material for: Pathogenicity Classification of TARDBP Variants of Uncertain Significance: An Integrative Clinical Characterization and Functional Validation
Source: Cells. 2026 Jul 8;15(14):1232. doi: 10.3390/cells15141232 (PMC13407010; doi:10.3390/cells15141232)

Supplementary Material

Table S1: ACMG-Classified *TARDBP* Variants with Known Pathogenicity

Exon	Variants	Pathogenicity classification (evidence)
2	c.36C>T (p.Asn12Asn)	LB (PM2+BP6+BP7)
2	c.198T>C (p.Ala66Ala)	B (BA1+BS2+BP6+BP7)
6	c.774C>G (p.Ser258Ser)	LB (PM2+BP6+BP7)
6	c.943G>A (p.Ala315Thr)	P (PS3+PS4+PM1+PM2+PP1+PP2+PP5)
6	c.991C>A (p.Gln331Lys)	LP (PM1+PM2+PP2+PP3+PP5)
6	c.1009A>G (p.Met337Val)	P (PS3+PS4+PM1+PM2+PP2+PP3)
6	c.1144G>A (p.Ala382Thr)	P (PS3+PS4+PM1+PM2+PP2)

Figure S1: Control assays with variants of established pathogenic classification. (A) HEK293T cells were transiently transfected with wild-type or mutant TDP-43 plasmids for 48 h and observed under confocal microscopy. Nuclei are visualized with blue fluorescence; GFP (green fluorescence) indicates successful transfection; TDP-43 is labeled with red fluorescence. Scale bar: 5 μm. (B) Quantitative comparison of the percentage of aggregate-positive cells between the wild-type and mutant variants (C) Quantitative comparison of the percentage of cells with aberrant nuclear translocation between the wild-type and mutant variants. ns, not significant. * $p < 0.05$, ** $p < 0.01$.

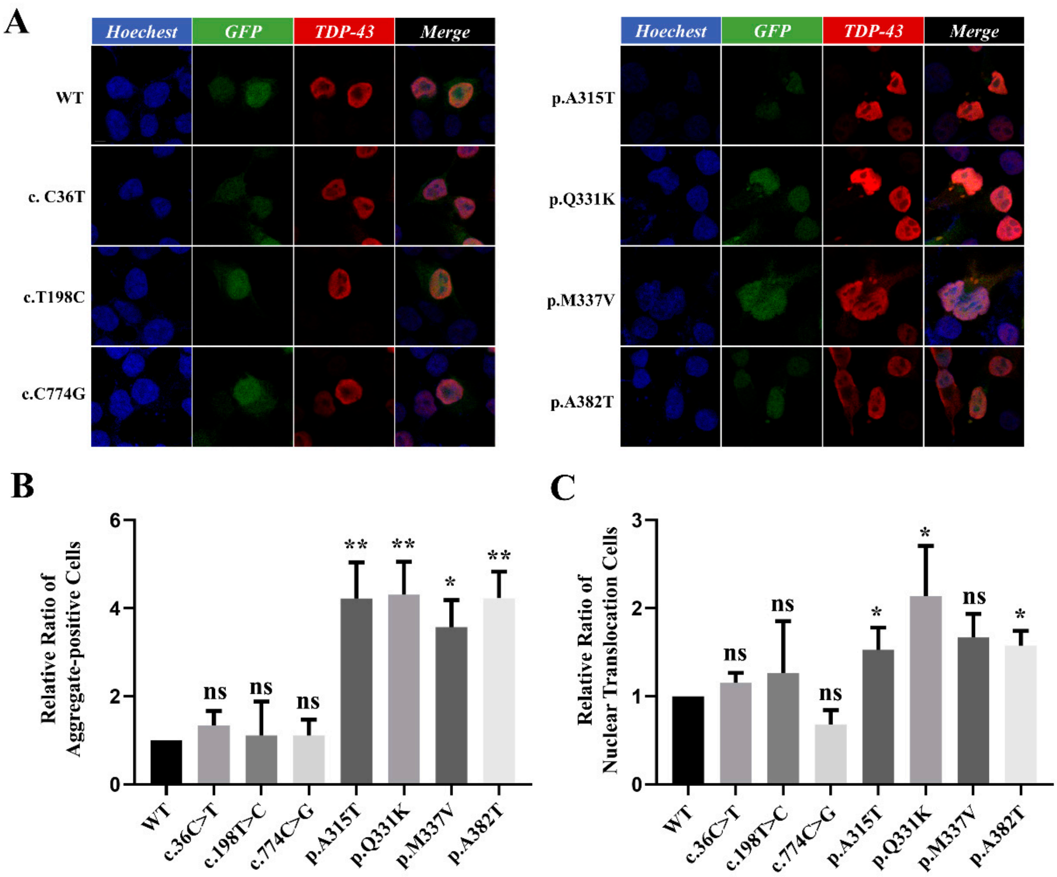

We selected four well-established B or LB and four well-established P or LP TARDBP variants as controls (Table S1). Our results consistently demonstrated that known P or LP variants exhibited significantly increased aggregate formation compared with wild-type TDP-43, whereas no statistically significant difference was observed for known B or LB variants.

We considered increased TDP-43 aggregation as evidence of functional impairment due to GOF, and increased aberrant nuclear-cytoplasmic translocation as evidence of functional impairment due to LOF. According to the recommendations from the ClinGen Sequence Variant Interpretation (SVI) Working Group [38], the odds of pathogenicity are 3.0 for GOF evidence and 2.3 for LOF evidence, both qualifying as PS3_supporting evidence.

Figure S2: Expression of TDP-43 Protein Carrying p.T88I and p.L248* Variants. HEK293T cells were transiently transfected with pEGFP-TDP-43 vectors encoding wild-type or mutant TDP-43, followed by extraction of total cellular protein and immunoblot analysis.

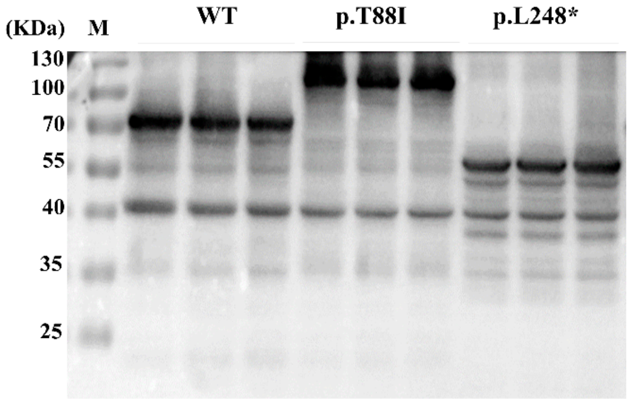

Figure S3: Expression Levels of Autophagy Markers LC3 and p62/SQSTM1 in Cells Expressing TDP-43 Carrying the p.A382S Variant. (A) HEK293T cells were transiently transfected with pEGFP-TDP-43 vectors encoding wild-type or p.A382S TDP-43, followed by extraction of total cellular protein and immunoblot analysis. (B) Quantitative analysis of protein levels. Data are presented as mean ± standard deviation (SD). ns, not significant. ****p < 0.0001 vs. wild-type.

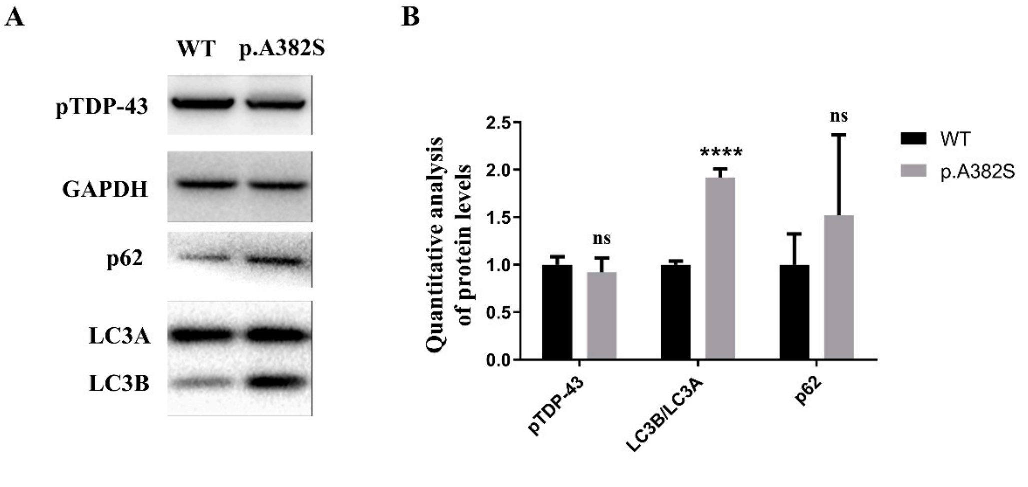

Figure S4: G3BP1 Expression in Skin Fibroblasts. Immunofluorescence staining of fibroblasts carrying *TARDBP* p.G298V or p.S375G variants and control fibroblasts. Cells were labelled with an antibody against TDP-43 (red). Scale bars: 10 um. NC, normal control.

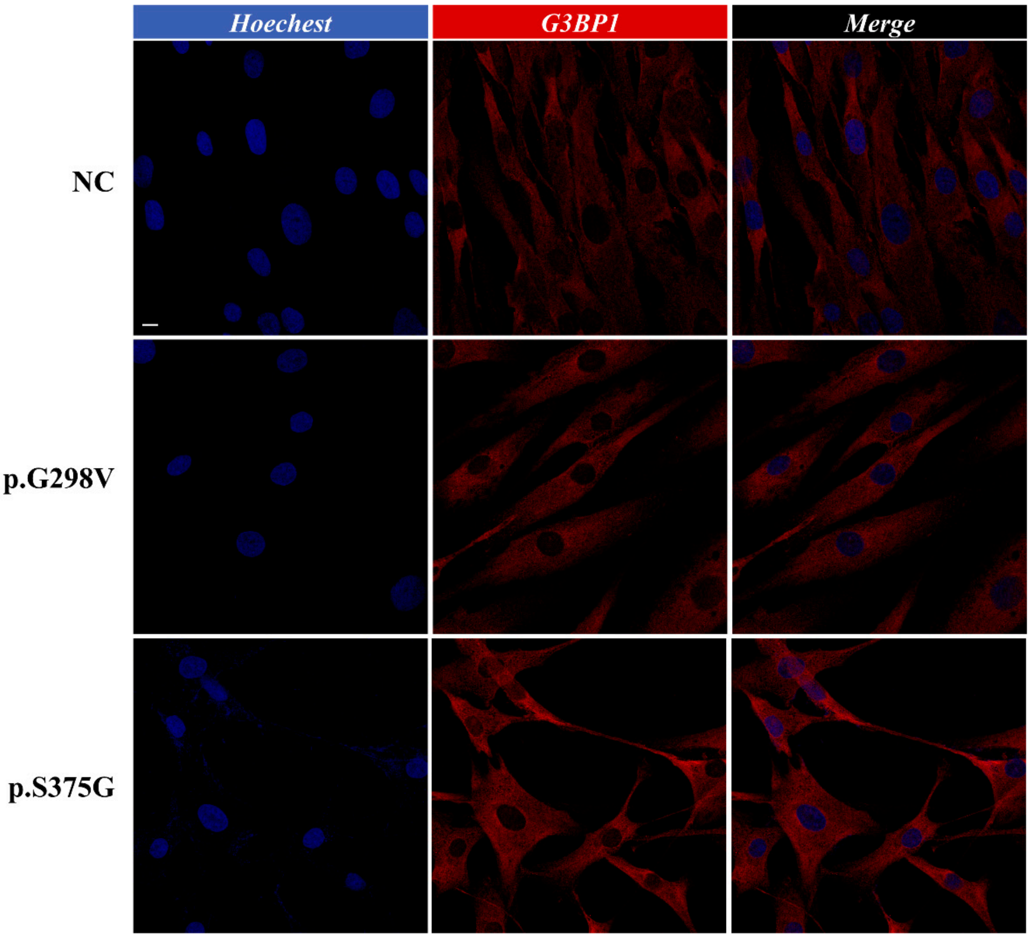

Supplement: Supplementary file 1 [file cells-15-01232-s001.zip › cells-4321901-supplementary.pdf]
